# Strategies to include prior knowledge in omics analysis with deep neural networks

**DOI:** 10.1016/j.patter.2025.101203

**Published:** 2025-03-14

**Authors:** Kisan Thapa, Meric Kinali, Shichao Pei, Augustin Luna, Özgün Babur

**Affiliations:** 1 Computer Science Department, University of Massachusetts Boston, 100 Morrissey Boulevard, Boston, MA 02125, USA; 2 Developmental Therapeutics Branch, Center for Cancer Research, National Cancer Institute, NIH, 9000 Rockville Pike, Bathesda, MD 20892, USA; 3 Computational Biology Branch, National Library of Medicine, NIH, 9000 Rockville Pike, Bathesda, MD 20892, USA

**Keywords:** deep learning, multi-omics, biological prior knowledge, graph neural networks

## Abstract

High-throughput molecular profiling technologies have revolutionized molecular biology research in the past decades. One important use of molecular data is to make predictions of phenotypes and other features of the organisms using machine learning algorithms. Deep learning models have become increasingly popular for this task due to their ability to learn complex non-linear patterns. Applying deep learning to molecular profiles, however, is challenging due to the very high dimensionality of the data and relatively small sample sizes, causing models to overfit. A solution is to incorporate biological prior knowledge to guide the learning algorithm for processing the functionally related input together. This helps regularize the models and improve their generalizability and interpretability. Here, we describe three major strategies proposed to use prior knowledge in deep learning models to make predictions based on molecular profiles. We review the related deep learning architectures, including the major ideas in relatively new graph neural networks.

## The bigger picture

Machine learning models, including those based on deep learning, are capable of processing complex datasets to generate a variety of predictions. Typically, these models rely solely on the provided data (often numeric matrices disconnected from their original context) as inputs to generic algorithms that overlook domain-specific knowledge that could enhance their predictive performance. In biology, machine learning is frequently used to convert molecular measurements into predictions, such as determining prognosis or drug response. Researchers have observed that incorporating existing biological knowledge about molecular interactions can enhance both the accuracy and interpretability of these predictions. However, integrating data and knowledge through deep learning is not straightforward. We review this topic (i.e., implementation and interpretation strategies) in the context of molecular biology.

## Teaser

The authors review methods to improve deep learning models for analyzing molecular data by integrating biological prior knowledge. This review explores several strategies, including transforming raw omics data into structured features using gene sets, modifying neural network architectures to reflect biological relationships, and applying GNNs to incorporate known molecular interactions.

## Background

High-throughput molecular profiling technologies—categorized into genomics, transcriptomics, proteomics, and metabolomics (“omics” as an umbrella term, see Box 1)—have accelerated biological research, but also brought the challenge of interpreting them. Each omic modality brings a different perspective. These data are typically very high dimensional and tightly interrelated. When integrated, they provide a holistic view, but their complexity requires sophisticated analysis methods.

Box 1 **Molecular profiles**

**Figure 1:**
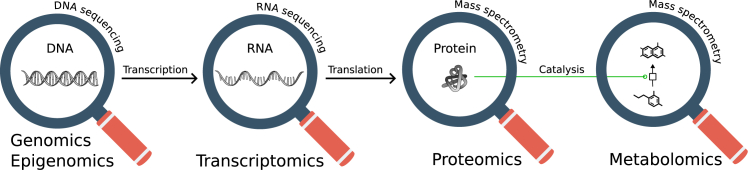
A high-level overview of high-throughput molecular profiling technologies in molecular biology

High-throughput molecular profiling technologies accelerated molecular biology research in the past decades. They provide a comprehensive view of the contents of cells in different modalities, each modality focusing on one type of biomolecule (Figure 1).

The common suffix—omics—is used as an umbrella term, and multiple modalities together are often called “multi-omics.”

Some common types of molecular profiling modalities include:

• *Genomics:* identification of the DNA sequence including genes and non-coding sequences. Compared with the reference genome, many genomic alterations can be detected, such as single-nucleotide variations, deletions, inversions, copy-number alterations, etc.
• *Epigenomics:* measurement of DNA methylation and histone modifications, which regulate genomic accessibility and gene expression.
• *Transcriptomics:* measurement of RNA molecules such as mRNA, miRNA, or non-coding RNA. Typically, RNA sequencing is used. Depending on the technology and preference, the detected sequences can be short (∼50 bp) or long (>100 bp). Long read length may be used to detect splice variants, while shorter reads tend to be more accurate.
• *Proteomics:* measurement of proteins and their modifications. Mass spectrometry is commonly used in proteomics to detect peptides generated by breaking down proteins. These peptides may be filtered by post-translational modifications, such as phosphopeptides, which provide a measure for protein modification. Another proteomics technology is reverse-phase protein array where antibodies on microchips are used to detect proteins.
• *Metabolomics:* measurement of small-molecule metabolites through mass spectrometry.

These profiling modalities originally invented for “bulk” analysis, meaning cell contents in a sample are mixed and their average is measured. With the technological advancements, new methods emerged to measure individual cell contents, dubbed as “single-cell omics.”^1^

A neural network (see Box 3) is a method in machine learning that can deal with this complexity due to its multi-layer structure and non-linearity. Neural networks are commonly used for omics analysis in tasks such as predicting phenotypic features, disease subclass/prognosis, or drug response values. This approach, however, comes with a major challenge: if the number of training samples is not sufficiently high, then complex models will overfit the data, meaning that they will learn the random fluctuations rather than the actual signal. In these cases, the model will not generalize well to the unseen data, rendering such models useless. In molecular profile analysis, the sample size is typically much smaller than the feature size (number of measurements), therefore, controlling the model complexity (also known as regularization) is essential for a successful analysis.

A standard regularization method for neural networks is to penalize the non-zero model parameters (weights) and force the learning algorithm to keep only the most useful ones. This is equivalent to reducing the number of connections (arrows) in Figure 4 in Box 3 for a simpler structure. While useful, this approach may not be sufficient because even with reduced connections, the model search space can be very large, challenging the learning algorithms to converge to a generalizable model. A solution is to reduce the search space with the help of prior biological knowledge. Here, the idea is that biological molecules interact and work together to determine the downstream events; therefore, considering such functional groups of molecules together instead of evaluating them individually may provide a simplified clearer signal.

Biological molecule interactions can be modeled at different complexities, such as gene sets, undirected networks, directed networks, or process models (see Box 2). These models capture how the molecules are organized, interact, and relate to known biological events or structures. More complex models provide molecular reactions in detail. Wysocka et al.^2^ provide a systematic review of biologically informed deep learning models specific for cancer, emphasizing the integration of prior knowledge in multi-omics analysis. Their work highlights the role of prior knowledge in three key areas: enriching input data, structuring deep learning architectures, and interpreting model outputs, all of which contribute to enhancing the overall biological interpretability of the models. Ballard et al.,^3^ and Kang et al.^4^ provide overviews of deep learning approaches for multi-omics data analysis, but these are more general, without the prior knowledge focus. Here, we review the strategies for incorporating prior knowledge into deep learning frameworks for effective regularization and increasing the performance, generalizability, and interpretability. We classify these strategies into three major categories: (1) using prior knowledge for input transformation, (2) making neural network structure resemble the connections in prior knowledge, and (3) using graph neural networks (GNNs). We summarize these in the next section and provide a list of referenced methods in Table S1.

Box 2 **Biological prior knowledge**

**Figure 2:**
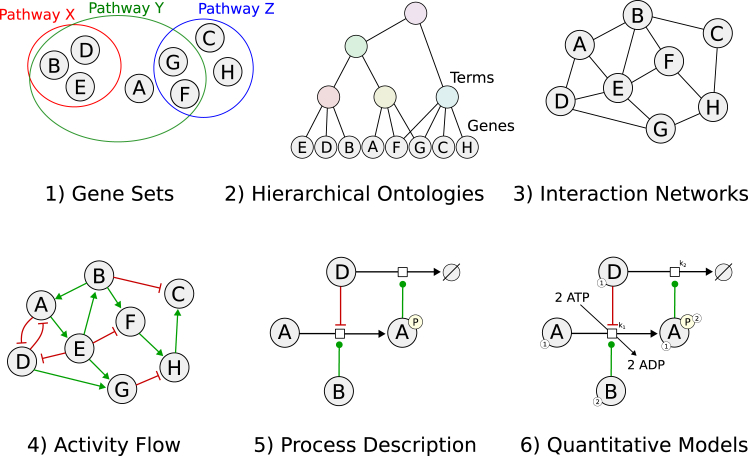
Six levels of prior knowledge of how biomolecules are related Figure modified from Sommers et al.^5^

The prior knowledge of how biological macromolecules are related is captured in various forms. We recently contributed to a study where we categorized this knowledge according to their complexity levels^5^ (Figure 2).

1. *Gene sets:* groups of genes that share common biological functions, or any other properties, often curated from databases and literature. Some resources: MSigDB,^6^ GeneSetDB,^7^ etc.
2. *Hierarchical ontologies:* models of biological phenomena as terms, relations between terms, and associated genes. These models are generally carefully curated as a directed acyclic graph, giving it a hierarchical structure. Common resources: Gene Ontology,^8^^,^^9^ KEGG BRITE,^10^ etc.
3. *Interaction networks:* binary and undirected relations between genes or proteins. PPI networks are the most popular form of this class, where an edge means two proteins physically interact. Another example is genetic interaction networks, where an edge represents the synthetic lethality of two genes. Some resources: BioGRID,^11^^,^^12^ IntAct,^13^ HPRD,^14^ etc.
4. *Activity flow:* directed (and sometimes signed) relations between proteins. These types of networks are either curated from literature, or they are derived from more detailed models. Common resources: Pathway Commons in SIF form,^15^ CausalPath prior network,^16^^,^^17^ Reactome functional interaction network,^18^ etc.
5. *Process description:* process-centric models of molecular events. Processes are first-class entities in these models (small squares in Figure 2) where biomolecules act as inputs, outputs, or controllers. Details such as protein modifications, molecular complexes, or cellular locations are often included. In addition, subgraphs of these models are often identified and given descriptive names, such as “Signaling by EGFR,” referred to as “pathways.” Pathway names are organized in a hierarchical structure, top-level pathways mapping to larger subgraphs that contain multiple lower-level pathways. Some resources: Reactome,^19^ KEGG,^20^ BioCyc,^21^ PantherDB,^22^ PhosphoSitePlus,^23^ Pathway Commons in BioPAX form,^24^ etc.
6. *Quantitative models:* this is the most detailed representation of biological pathways where the processes are fully characterized with stoichiometry of molecules and reaction rate constants. These models have enough details for a computer simulation of molecular events; however, they are highly context specific, rare, and generally built on a small scale. BioModels database is an example resource.

In the literature, it is common to see these prior knowledge modalities converted into each other through reductions. For instance, hierarchical ontologies can be reduced to gene sets by propagating gene associations upward and then ignoring relations between terms. Or a network (directed or undirected) can be reduced to gene sets by taking neighborhoods of nodes. A directed network can be used in an undirected manner by ignoring edge directions. KEGG and Reactome resources are often used as a hierarchical ontology by keeping the pathway name hierarchy but reducing the process model subgraphs into gene sets.

In the scientific literature, the term “pathway” could refer to any prior knowledge modality, including gene sets. Since this creates confusion, we reserve this word for subgraphs of process description models. In this review, we specifically refrain from referring to gene sets as pathways.

## Strategies

### Using prior knowledge to generate input features to the learning algorithm

Instead of using molecular profiles directly as inputs to the learning algorithm, transforming them into structures that capture relations and functional groups of genes has been shown to improve the clarity of the signal, and increase the predictive performance. We identify two major approaches in the following subsections that use prior knowledge to transform the input. The first idea is to use gene set/pathway enrichment scores. The second idea is to convert molecular profiles into two-dimensional (2D) images where gene values are mapped on certain pixels such that similar genes map to close pixels. This generates regions on the image that correspond to functional groups, which are subsequently analyzed by methods specialized to images.

#### Using gene set enrichment scores as inputs to neural network

**Figure 3:**
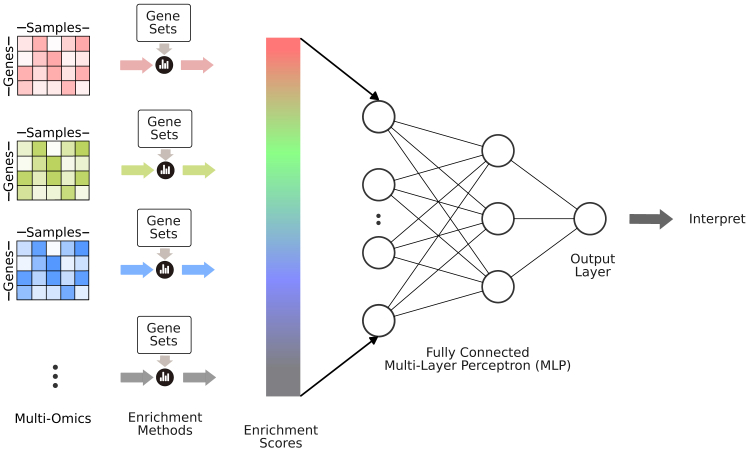
Transforming inputs into enrichments Gene set enrichment scores are calculated (using GSEA,^25^ ssGSEA,^26^ NetPEA,^27^ etc.) and used as input features to deep learning models.

Gene sets are groups of genes that share common properties such as being part of the same signaling pathway (e.g., members of the Wnt pathway), sharing a cellular location (e.g., nuclear proteins), functioning in the same cellular process (e.g., DNA repair genes), etc. MSigDB^6^ is a popular and comprehensive resource for defined gene sets in human and mouse. Gene set enrichment analysis (GSEA) aims to translate individual gene values to scores for cellular processes/pathways. Suppose the activity of those cellular processes has predictive value for the current task. In that case, their gene set enrichment score may provide a consolidated, less noisy signal to the prediction algorithm compared with individual gene values. In addition, if the number of gene sets in the analysis is less than the number of total genes, then this transformation reduces input dimensions, and this helps the learning algorithm to be more efficient (Figure 3).

GSEA was first described by Subramanian et al.^25^ Many variations have since been published. DeepCC^28^ uses a version of GSEA to transform gene expression data, and subsequently feed it to a neural network (see Box 3) for cancer subtype classification. Their GSEA step is as follows:

1. For each sample and each gene, calculate the log fold change value by taking the log of the ratio of the gene’s value to the gene’s average expression across samples.
2. For each sample, rank the genes by log fold change values in descending order.
3. For a given ranking of N genes and a given gene set *S*, calculate the enrichment score *e* as

(Equation 1) $$e\left(S\right)=\max_{1\leq i\leq N}\left|P_{\text{hit}}\left(S,i\right)-P_{\text{miss}}\left(S,i\right)\right|$$

where

• *i* is a location on the gene ranking;
• $P_{\text{hit}}\left(S,i\right)=\frac{\left(\left|\{s:s\in S\;\wedge \;\text{rank}\left(s\right)\leq i\}\right|\right)}{\left|S\right|}$, which is the proportion of *S* members at location *i* or smaller to |S|;
• $P_{\text{miss}}\left(S,i\right)=\frac{\left|\{s:s\in \bar{S}\;\wedge \;\text{rank}\left(s\right)\leq i\}\right|)}{\left|\bar{S}\right|}$, which is the proportion of $\bar{\text{S}}$ members at location *i* or smaller to $\left|\bar{S}\right|$;
• rank(s) is the location of gene *s* on the ranking;
• $\bar{S}$ is the complement set of *S*, contains the genes that are not in S;
• |S| is the cardinality of *S*, which is the number of genes in S.

Box 3 **Neural network basics**

**Figure 4:**
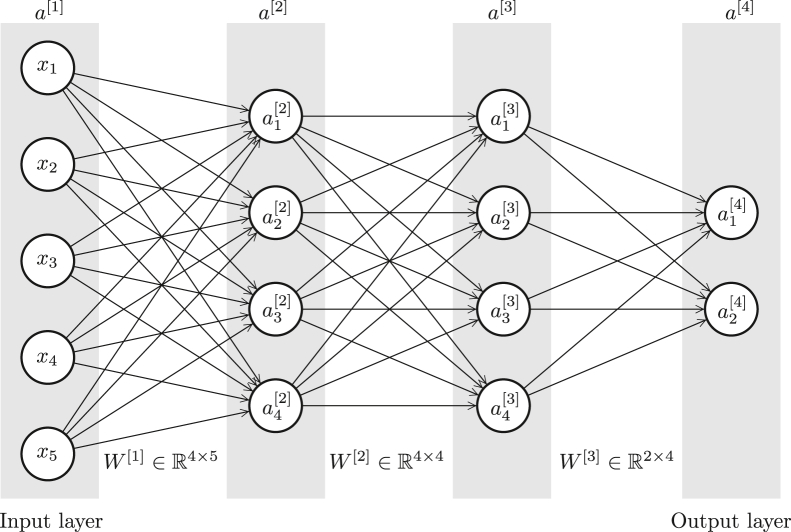
A sample MLP This MLP has an input size of 5, an output size of 2, and two hidden layers with a size of 4. Each circle on the diagram, except the circles in the input layer, represents a perceptron.

A multi-layer perception (MLP), or simply a feedforward neural network is composed of calculating units (or perceptrons) organized in layers (Figure 4). Each perceptron is responsible for processing an input vector by taking its inner product with a weight vector and applying a non-linear transformation to generate its output.

$$\text{output}\;\text{of}\;a\;\text{perceptron}=\sigma \left(\left[\begin{array}{l} x_{1}\\ x_{2}\\ x_{3}\\ \vdots \end{array}\right]\cdot \left[\begin{array}{l} w_{1}\\ w_{2}\\ w_{3}\\ \vdots \end{array}\right]+b\right)=\sigma \left(x_{1}w_{1}+x_{2}w_{2}+x_{3}w_{3}+\cdots +b\right)$$

where σ(·) is traditionally the sigmoid function.

$$\text{sigmoid}\left(x\right)=\frac{1}{1+e^{-x}}$$

The first layer of a neural network is called the input layer, and the last layer is called the output layer. The output layer may have one or more perceptrons depending on the desired dimensionality of the network output. Other layers are called hidden layers, and their size (number of perceptrons in them) is subject to optimization. A perceptron on a specific layer takes its inputs from the outputs of previous layer perceptrons. This relationship is indicated by arrows in Figure 4.

$$a_{i}^{\left[l+1\right]}=\sigma \left(\left[\begin{array}{l} a_{1}^{\left[l\right]}\\ a_{2}^{\left[l\right]}\\ a_{3}^{\left[l\right]}\\ \vdots \end{array}\right]\cdot \left[\begin{array}{l} w_{i1}^{\left[l\right]}\\ w_{i2}^{\left[l\right]}\\ w_{i3}^{\left[l\right]}\\ \vdots \end{array}\right]+b_{i}^{\left[l\right]}\right)=\sigma \left({a^{\left[l\right]}}^{T}w_{i}^{\left[l\right]}+b_{i}^{\left[l\right]}\right)$$

Values of perceptrons at layer l+1 are collectively calculated as below.

$$a^{\left[l+1\right]}=\sigma \left(W^{\left[l\right]}a^{\left[l\right]}+b^{\left[l\right]}\right)$$

The $W^{\left[l\right]}$ matrix is used to transform the values at layer *l* into the values at layer l+1. Its number of rows equals the size of layer l+1, and its number of columns equals the size of layer *l*. Each row of the matrix corresponds to the weight vector of a perceptron at layer l+1, meaning $W_{ji}^{\left[l\right]}$ represents the arrow from the $i^{\text{th}}$ perceptron of layer *l* to the $j^{\text{th}}$ perceptron of layer l+1.

The term *deep learning* refers to a neural network with multiple hidden layers. Modern deep learning architectures prefer the ReLU function over the sigmoid function in hidden layers, which establishes non-linearity by replacing negative values with 0.

ReLU(x)=max(0,x)

This score reflects the degree to which a gene set is overrepresented at the top or bottom of the ranked list of genes. Enrichment scores are used after standardization (subtract mean, divide by standard deviation).

Tang and Gottlieb^29^ predict drug sensitivity of cells based on multi-omics using a similar strategy. They describe a deep learning system where gene expression data are transformed into gene set enrichment scores through the method named ssGSEA,^26^ which is a variation where $P_{\text{hit}}$ does not treat gene set members equally but weighs them according to their alteration level in a sample. It also uses the sum function instead of the max function.

To calculate gene set scores on copy-number variation (CNV) and mutation data, Tang and Gottlieb take another approach that is arguably better suited to categorical data (genes are either altered or not altered). They use a network-based iterative algorithm called NetPEA,^27^ which uses a protein-protein interaction (PPI) network to assess how the altered genes are related (or close) to the defined gene sets. NetPEA runs a Random Walk with Restart procedure^30^ using the altered genes as seeds on the network. The algorithm calculates the probability of the random walk to start from a seed and end up at any specific node in *n* iterations, formulated as

(Equation 2) $$S^{n}=\left(1-p\right)MS^{n-1}+pV$$

where

• *V* is the vector of initial node probabilities ($V_{i}=\frac{1}{\left|\text{seeds}\right|}$ if *i* is a seed node, otherwise $V_{i}=0$);
• *p* is the restart probability where restart means jumping back to a seed node, set to 0.5 in this case;
• *M* is the transition probability matrix derived from the PPI network ($M_{ij}=\frac{1}{\left|N\left(i\right)\right|}$ if nodes *i* and *j* are connected, where |N(i)| is the number of neighbors of node *i*, $M_{ij}=0$ otherwise);
• ${\;S}^{n}$ is a vector representing probabilities of being on each node after *n* rounds of iteration where $S^{0}=V$.

The value of *n* is decided by the convergence of the $S^{n}$ vector. After probabilities are calculated for each node, a gene set score is determined as the average probability of its member genes.

Alongside the transformed omics, their system takes drug-related information as input, such as Morgan fingerprint of the drug and the gene set scores of the known drug targets (using drug targets from GDSC dataset^31^ instead of the altered genes in the NetPEA algorithm). Such input enables the model to predict drug sensitivity for never-seen drugs based on their structural and target similarity to the drugs in the training.

There are many other approaches in the literature for generating gene set scores. For instance, the method PathME^32^ uses an autoencoder (see the following sections) to generate gene set scores. Any of these methods can be used to transform omics into neural network inputs. A comprehensive review of gene set enrichment methods is beyond our scope and can be found in other reviews.^33^

#### Converting molecular profiles to a 2D image and feeding it to a convolutional neural network

**Figure 5:**
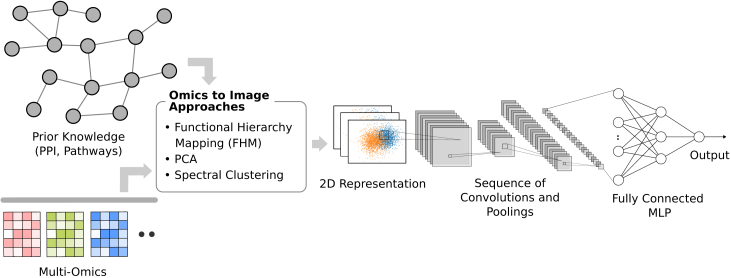
Transforming inputs into images The transformation is performed using functional hierarchy mapping, PCA, or spectral clustering. These 2D images are fed to convolutional neural networks for prediction tasks.

Convolutional neural networks (CNNs) (see Box 4) are specialized structures for image analysis. They are designed to recognize shapes, colors, patterns, and other image properties. Their success in image analysis encouraged researchers to convert their non-image data into an image and process with a CNN. For this strategy to work, the image must encode information in recognizable shapes/regions. Here, we identify three main approaches that convert molecular profiles into images using prior knowledge (Figure 5). Each approach uses a different prior knowledge modality to guide the formation of regions on the image, which are colored by the related gene values so that a CNN can pick up their consolidated signal. These methods are (1) functional hierarchy mapping guided by a hierarchical ontology, (2) principal-component analysis (PCA) guided by gene sets, and (3) spectral mapping guided by an undirected network such as PPIs.

Box 4 **Convolutional neural networks**

A convolutional neural network (CNN) is an extension to MLP, developed specifically for processing image inputs. Unlike the fully connected layers of an MLP, this architecture uses convolutional layers where each perceptron at layer l+1 takes input from only a small portion of the perceptrons at layer *l* that are geometrically close to each other.

The convolution of two equal-sized matrices means multiplying the corresponding elements and summing them to generate a single number.

**Figure:**
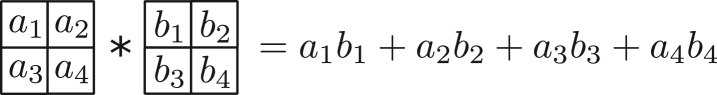

When the first matrix is larger (the image) and the second matrix is smaller (kernel, or filter), the result would be another matrix. In that case, convolution means the same operation is applied on submatrices of the larger matrix, each submatrix generating a value whose location in the result is determined by the location of the source submatrix.

**Figure:**
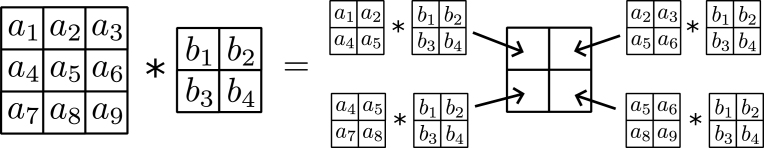

A CNN takes an image as an input, and it has matrices (from images) at the convolutional layers (as opposed to vectors). Values in a convolutional layer are generated by applying convolution to the previous layer and applying a non-linear transformation to the result, as in the following example.

**Figure:**
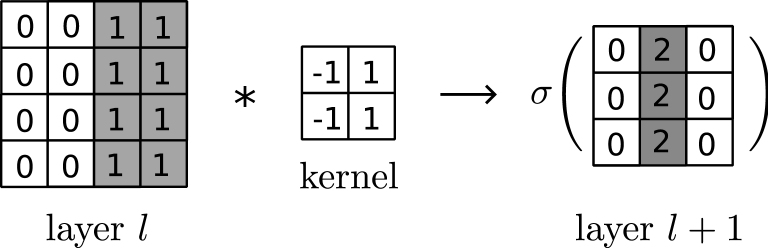

Here, the kernel recognizes light-to-dark transition points (or edges) on the image. CNNs typically use multiple kernels with learnable weights at each layer, and each kernel can specialize to recognize a distinct image feature and collectively generate a stack of images for the next layer. The non-linear function σ(·) is typically the ReLU function.

CNNs contain multiple convolutional layers. The output of the last convolutional layer is flattened (converted into a vector) and processed with an MLP for the task at hand, such as classification. While the CNN architectures contain many other features and details, those are beyond the scope of this review. The use of kernels lets CNNs recognize localized features, encoded by proximate pixels. Repeated convolutional layers help connect local features to recognize global features.

**Figure 6:**
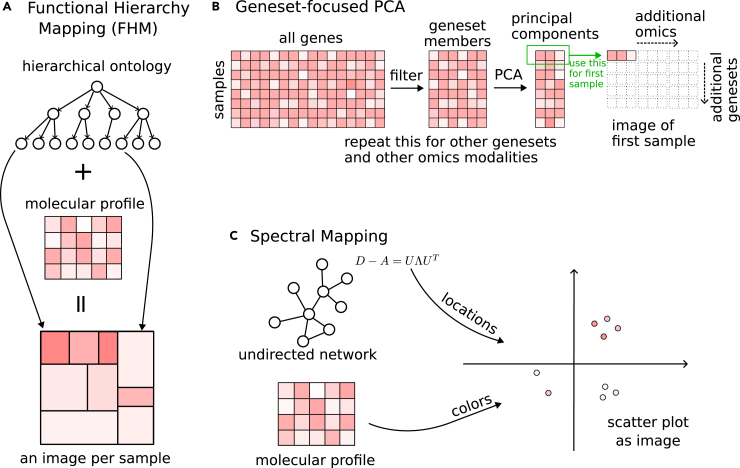
Overview of three approaches for converting molecular profiles into images (A) Functional hierarchy mapping, (B) gene set-focused PCA, and (C) spectral mapping.

Functional hierarchy mapping uses a hierarchical ontology to map molecular profiles to a rectangular image (Figure 6A). López-García et al.^34^ use the treemapping algorithm^35^ to generate such an image of gene expression data based on KEGG BRITE ontology,^20^ which recursively divides the image into subrectangles following the nesting structure between the terms of the hierarchy, and color codes a rectangle with the average expression of a term’s associated genes. They apply this approach to lung cancer gene expression data in TCGA,^36^ process the generated images with a CNN, and predict lung cancer progression. Since the number of lung cancer samples is not very high, the authors formulate a transfer-learning approach to increase the prediction performance: they process expression data from all cancer types, convert them to images, and train a CNN to predict survival; then they fine-tune this model to predict the progression of lung cancer. This approach benefits from the larger sample size in the pan-cancer dataset, solves a different but similar problem to generate a model, and then uses the model parameters as a better starting point for the actual problem.

Wang et al.^37^ generate two images for each gene expression sample using two different hierarchies: one image is based on KEGG BRITE,^10^ and a second one is based on KEGG pathways hierarchy. To prepare an image, they sort the low-level terms in the ontology so that related terms stand close and also sort the member genes of a term with their average expression across samples. Sorted values are then placed on a rectangular grid. They concatenate these two images and predict lung cancer long-term survival using a CNN. Their system also uses clinical data such as age and tumor stage. Since the clinical data are not part of the generated images, they concatenate it to the output of the convolutional layers and feed it to the fully connected layers at the end of the architecture.

Another approach uses PCA for omics-to-image transformation (Figure 6B). PCA is traditionally used for reducing input data dimensions in machine learning with minimal information loss. For instance, a gene expression matrix with 20,000 dimensions (genes) may be reduced to 200 dimensions (principal components) while still preserving half of its original variation. Such reduction helps with computational efficiency and may alleviate some problems related to the high dimensionality of the data, such as overfitting. The PathCNN^38^ method uses PCA in a gene set-centric way where gene sets are derived from KEGG pathways.^20^ Instead of applying PCA on the whole data, it generates submatrices of the data by filtering in the genes using a gene set, applies PCA to each submatrix, and takes the first three principal components to represent the gene set for each sample. This is somewhat similar to transforming the data using gene set scores, but instead of a single score, PathCNN represents the state of each gene set with three values coming from principal components. The authors apply this to multi-omics (gene expression, CNV, and DNA methylation data, see Box 1) of glioblastoma patients, generate a 9D representation of each gene set (three values per omics modality) for each patient, arrange these into a 9-pixel wide image for each patient where each row corresponds to a gene set, and predict long-term survival by using these images as input to a CNN.

The third approach we mention for generating image representations of molecular profiles uses spectral graph theory^39^^,^^40^ (Figure 6C). It takes a connected undirected graph (typically a PPI network) and maps each node on the graph to a point in 2D space. Molecular profile values of genes are represented on these points with colors to generate an image of the profile. Nodes that are related and have similar connections on the graph map to close points, while nodes that are distant on the graph map to distant points. For instance, a large protein complex, represented with a clique on the PPI network would likely map to close pixels on the image, generating a region about that complex on the image.

Spectral mapping uses the Laplacian of the graph.

(Equation 3) L=D−A

where

• *A* is the adjacency matrix of the graph ($A_{ij}=1$ if nodes *i* and *j* are connected; $A_{ij}=0$ otherwise);
• *D* is the diagonal degree matrix, where, $D_{ii}=\sum_{j}A_{ij}$.

Then it performs eigenvalue decomposition on the Laplacian matrix *L* to obtain its eigenvectors and eigenvalues.

(Equation 4) $$L=U\Lambda U^{T}$$

where

• *U* is the matrix that has eigenvectors in its columns;
• Λ is the diagonal matrix of eigenvalues, sorted in ascending order ($\Lambda_{11}=0<\Lambda_{22}<\Lambda_{33}<\cdots$).

The first eigenvalue always comes out as 0 and the corresponding eigenvector is a constant vector, carrying no information. Spectral mapping uses the next 2 eigenvectors that correspond to the next 2 non-zero eigenvalues ($\Lambda_{22}$ and $\Lambda_{33}$) for the mapping. In other words, the second and the third columns of *U* provide coordinates for node locations, which generates a 2D embedding that carries information about the graph structure.

Matsubara et al.^41^ use spectral mapping for diagnosing lung cancer based on gene expression data from patients undergoing diagnostic bronchoscopy. They use the PPI network from the HINT database^42^ to guide the mapping. They reduce the generated images to 100×100 pixels by applying 1D convolutional filters and train a CNN for the diagnosis. Chuang et al.^43^ extend this work to predict cancer types in 11 cancer studies in TCGA. To focus on the most interesting features, they detect differentially expressed genes (DEG) between normals and tumors for each study and combine them. Then they build a PPI network by integrating 5 sources (BioGRID,^11^ DIP,^44^ IntAct,^13^ MINT,^45^ and MIPS^46^), and get an induced subgraph of the network using the DEGs (an induced subgraph of a group of nodes is the graph containing these nodes and the connections between them). They do a spectral mapping of DEG expression values using the PPI subgraph and train a CNN to recognize the cancer type or predict if it is benign.

### Structuring the neural network layers imitating the organization in prior knowledge

The second major strategy for including prior knowledge in deep learning is through structural modifications to the neural network (see Box 3) so that the network connections resemble the graph structure of prior knowledge. This is often applied to either a multi-layer perceptron (MLP) or an autoencoder.

#### Through modifications to MLP

**Figure 7:**
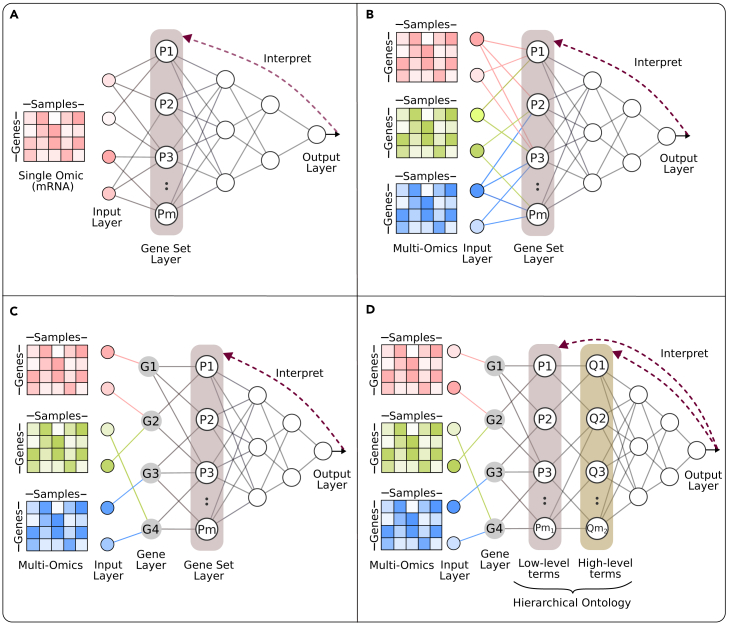
Illustration of approaches that imitate prior knowledge organization in the neural network architecture in four levels with increasing complexity (A) The first hidden layer represents gene sets (gene set layer). (B) Similar to (A), but multiple molecular profile types are used, meaning there are multiple input values per gene. (C) Similar to (B), but there is an additional layer (gene layer) between inputs and the gene set layer. The perceptrons in the gene layer represent genes: they are connected to an input node only if that input belongs to that gene. (D) Similar to the previous architectures, but a hierarchical ontology is used instead of plain gene sets, which is represented in multiple hidden layers.

Forcing MLP network connections to resemble the graph structure of prior knowledge may seem unintuitive because the neural network and prior knowledge have different objectives. There is no obvious reason for their structures to resemble each other in a successful model. To understand the appeal, let us start with the simplest version of this strategy: mapping the first hidden layer of an MLP to gene sets (Figure 7A).

Let $x\in R^{n}$ represent the input vector of molecular profile values, where *n* is the number of genes, and let each perceptron on the first hidden layer represent a gene set. Assume there are *m* gene sets, hence, *m* perceptrons in the first hidden layer. The gene set membership information can be encoded in the connections on the neural network, specifically between the input layer and the first hidden layer through the weight matrix $W^{\left[1\right]}\in R^{m\times n}$ such that $W_{ji}^{\left[1\right]}$ is a learnable parameter if gene *i* is a member of the gene set *j*. Otherwise, $W_{ji}^{\left[1\right]}$ is fixed at 0 (see Box 3 for neural network notation).

This approach is similar to using gene set enrichment scores as input. The difference is that we migrate gene set score calculation into the neural network, letting the learning algorithm decide which members of the gene set are more important and which ones it can ignore by adjusting the weights between input and gene set-related perceptrons. This is a more adaptive way of converting gene values into gene set scores, which may improve prediction accuracy. But adaptiveness comes with the cost of interpretability: since the weights between a gene set perceptron and the inputs of its member genes are free to change during learning, there is no guarantee that the perceptron will preserve the gene set’s biological meaning. Nevertheless, this possibility is often not discussed, and the influence of these perceptrons on the results is interpreted as the importance of the biological mechanism of the gene set to the prediction.

The methods PASNet,^47^ Cox-PASNet,^48^ PAGE-Net,^49^ PINNet,^50^ and MPVNN^51^ use this strategy (Figure 7A) to predict cancer survival from TCGA gene expression data. The first three methods use gene sets in MSigDB derived from KEGG and Reactome pathways in the first hidden layer. MPVNN uses gene neighborhoods on directed signaling networks from KEGG as gene sets and names them “perturbations” of these genes, implying that a change in the neighborhood may perturb the activity of that gene, or the other way around, the perturbed activity of the gene may be the cause of the change in the neighborhood. Another method, PACL,^52^ applies the idea of the gene set layer to restricted Boltzmann machines (RBMs), which are similar to neural networks but are formulated using different mathematical concepts. Authors of PACL use gene sets to restrict connections of the hidden layer and use the RBM for subtyping glioblastoma and ovarian cancer gene expression profiles in TCGA.

This approach can also be used in a multi-omics setting (Figure 7B) where each gene has multiple input values from different omics types. To accommodate for multiple values, we modify the previous formulation as: $W_{ji}^{\left[1\right]}$ is a learnable parameter if input *i* is associated with a gene that is a member of the gene set *j*. The method DeepOmix^53^ uses this approach to predict survival from somatic mutations, copy-number alteration, gene expression, and DNA methylation data.

In the multi-omics setting, the interpretability of this approach can be improved by inserting a “gene layer” between the input layer and the gene set layer (Figure 7C). A perceptron on the gene layer represents a gene, which is connected to the input units that are related to that gene: $W_{ji}^{\left[1\right]}$ is a learnable parameter if input *i* is associated with gene *j*, otherwise, it is fixed at 0. The connections between the gene layer and the gene set layer (now the second hidden layer) are determined similarly: $W_{ji}^{\left[2\right]}$ is a learnable parameter if gene *i* is a member of gene set *j*. The existence of the gene layer makes it possible to measure the influence of gene-associated perceptrons on the output and interpret that as a measure of importance of that gene to the predicted feature. The method MiNet^54^ uses this approach to predict cancer survival from gene expression, copy-number alteration, and DNA methylation data with the help of gene sets from KEGG and Reactome pathways. Another method, consDeepSignaling,^55^ uses this approach to predict drug response of cell lines based on gene expression and CNV data, and gene sets from KEGG pathways. Their pipeline also takes known drug target information as input. The method treats drug targets as if this is another omics type where genes have a value 1 if they are a target of the drug, otherwise they have the value 0.

An alternative to using a gene set layer is to mimic connections of a hierarchical ontology (see Box 1) in the MLP hidden layers (Figure 7D). In that case, the second hidden layer represents low-level terms, which are associated with genes (represented on the first hidden layer), the third hidden layer represents the parent terms of the terms in the second hidden layer, and so on. If the layers *l* and l+1 represent ontology terms, then $W_{ji}^{\left[l\right]}$ is a learnable parameter if the term *i* is a child of term *j*, otherwise it is fixed at 0. This structure can also be used without a gene layer, or with single omics.

This approach is sometimes described as a visible neural network,^56^ suggesting that we can identify term-associated perceptrons that have a high influence on the output, and speculate that these ontology terms are most important to the predicted phenomena. The most popular hierarchical ontologies used for this architecture are Gene Ontology for detecting biological processes, molecular functions, and cellular components; and pathway name hierarchies derived from Reactome and KEGG pathways to detect pathway activities. The methods DCell,^56^ DrugCell,^57^ ParsVNN,^58^ BioVNN,^59^ P-NET,^60^ GenNet,^61^ Deep GONet,^62^ GCS-Net,^63^ SparseGO,^64^ MOViDA,^65^ and extension of GenNet^66^ all use a variant of this approach for a wide variety of tasks.

Even though the architectures we mention in this section are promoted for their interpretability, we would like to note that this should be done with caution. The learning algorithm does not see or care about the associations we make with the perceptrons, and it adjusts the weights to maximize the prediction performance, setting many of them to zero or very small values. This means the algorithm may choose to use only a small subset of the members of a gene set because the other members do not carry predictive information. A typical gene is associated with many gene sets and ontology terms, so this small subset can be in multiple common gene sets or terms. This means the learning algorithm can randomly choose any of these routes to send their signal to the output layer without considering which route represents the relevant biological mechanism. This point becomes clearer if we consider a case where the relevant biological mechanism is not represented among the gene sets. In this case, the predictive signal in the input will still find a way to propagate through the network using other associations of these inputs, leading to misinterpretation. Therefore, these interpretations should be considered as suggestions rather than detection.

#### Through modifications to autoencoders

An autoencoder is a neural network type designed to learn an encoding of input data, typically for dimensionality reduction or feature learning. It consists of two parts: an encoder that compresses the input data into a lower-dimensional latent space and a decoder that reconstructs the original data from this compressed representation. The encoder maps the input $x\in R^{n}$ to a latent representation $z\in R^{m}$ using a function $f_{e}\left(\cdot \right)$, where m<n. The decoder then reconstructs *x* from *z* using a function $f_{d}\left(\cdot \right)$.

(Equation 5) $$z=f_{e}\left(x\right),\text{and}\;x^{\prime }=f_{d}\left(z\right)$$

The first half of the neural network learns the function $f_{e}\left(\cdot \right)$ and the second half of the network learns the function $f_{d}\left(\cdot \right)$. The objective of the autoencoder is to minimize the differences between the original input *x* and the reconstructed output $x^{\prime }$ often using a loss function such as mean-squared error:

(Equation 6) $$L\left(x,x^{\prime }\right)={\left\|x-x^{\prime }\right\|}^{2}$$

**Figure 8:**
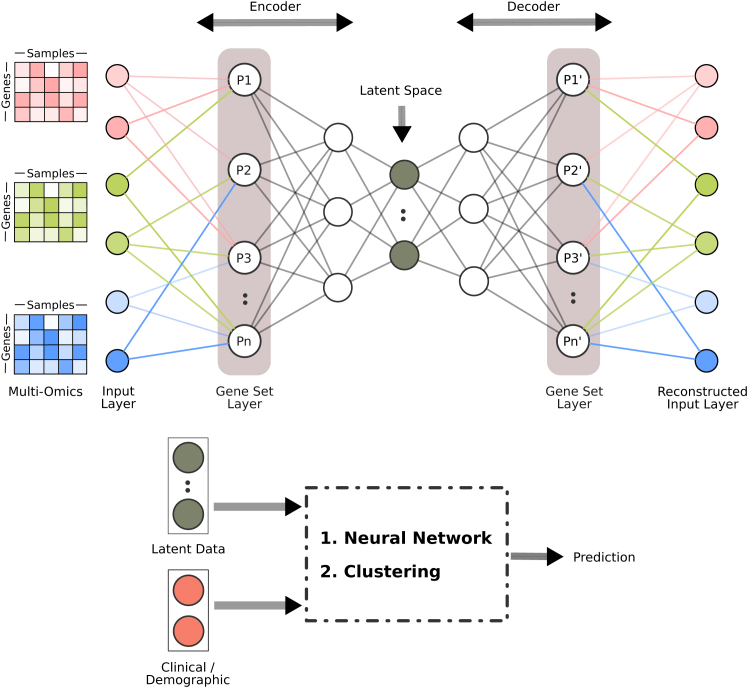
Illustration of incorporating prior knowledge to reduce the dimensionality of omics data using an autoencoder The autoencoder compresses the data into a lower-dimensional latent space, capturing the essential biological features. The latent space is then used as input to the neural network for downstream analysis.

Traditionally, autoencoder layers are fully connected. It is possible to integrate prior knowledge with autoencoders through a gene set layer similar to the previous approach (Figure 8). In this case, not only the first hidden layer but also the last hidden layer represents gene sets (the same gene sets). The connections between the input layer and the first gene set layer are mirrored in the connections between the second gene set layer and the output layer. By imitating the gene set structure in the hidden layers, the autoencoder passes the input through the filter of gene sets while encoding and decoding, making the latent space representation a compressed version of gene set scores. This potentially makes the latent space representation more useful for downstream tasks such as prediction or characterization, assuming the biological processes represented by the utilized gene sets are relevant to these tasks.

The methods GSAE,^67^ GOAE,^68^ VEGA,^69^ OntoVAE,^70^ and AUTOSurv^71^ use variants of this approach for tasks such as cancer survival prediction, clustering patients, or predicting biological pathway activity. VEGA uses the gene set layer only in the decoder, leaving the encoder fully connected. OntoVAE is designed similarly, but instead of adding a gene set layer, it incorporates a hierarchical ontology to the decoder through multiple layers.

Using a gene set layer (or multiple layers for a hierarchical ontology) can improve the interpretability and the relevance of the latent space representation of molecular profiles, but they may also be limiting. For instance, a relevant biological mechanism may be missing in the utilized gene sets because it has not yet been discovered. In that case, the inputs related to that mechanism may not be able to combine their signal properly if they are not part of any other common gene sets. To address this limitation, GOAE adds some fully connected perceptrons to the gene set layer. This is similar to defining new gene sets that include all genes as members, which puts the architecture between a fully connected autoencoder and a gene-set-informed one.

### Using graph neural networks

**Figure 9:**
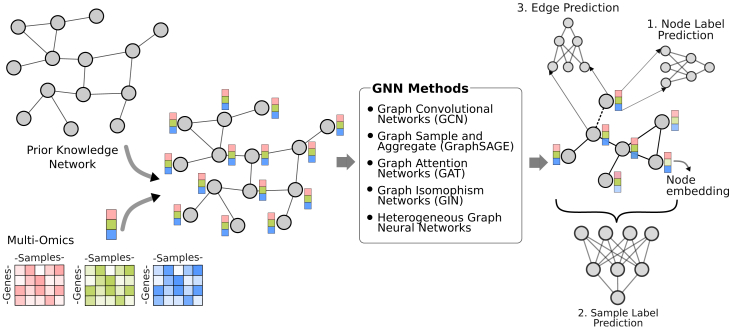
Overview of multi-omics analysis with GNNs The nodes on the prior knowledge network are associated with omics features, then fed into a GNN model. Most popular models are enumerated in the box: (1) graph convolutional networks (GCNs),^72^^,^^73^ (2) GraphSAGE,^74^ (3) graph attention networks (GATs),^75^ and (4) graph isomorphism networks (GINs).^76^ These GNN models learn new embeddings to use in downstream predictive tasks.

GNNs use a given graph structure as a guide to integrate features (inputs) associated with the nodes or edges of the graph. The rationale behind this is similar to spectral mapping: features of connected or close nodes on the graph are more likely to carry predictive information about the same phenomenon. When these features are consolidated, the signal becomes less noisy, more predictive. In molecular profile analysis, known PPIs, gene-gene interactions (GGIs), or gene regulatory networks (GRNs) can be used as a guide graph. Each omic modality would provide one feature associated with a node (gene or protein), making the size of the input feature vector of each node equal to the number of omics modalities in the analysis (Figure 9). Integration of these features depends on the specific GNN type.

A simple undirected graph G=(V,E) consists of a set of nodes $V=\left\{v_{1},v_{2},\ldots ,v_{n}\right\}$ and a set of edges $E=\left\{\left\{v_{i},v_{j}\right\}:v_{i}\;\text{and}\;v_{j}\;\text{are}\;\text{connected}\;\text{and}\;i\neq j\right\}$. The set of neighbors’ indices of a node is acquired by the function $N\left(i\right)=\left\{j:\left\{v_{i},v_{j}\right\}\in E\right\}$. To indicate a node and its neighbors, we use $N^{+}\left(i\right)=N\left(i\right)\cup \left\{i\right\}$. Each node $v_{i}\in V$ is associated with a feature vector $x_{i}$. GNNs operate on these graphs using message passing or neighborhood aggregation mechanisms to learn new feature vectors, which are subsequently used in predictions. The graph structure is often encoded in an adjacency matrix $A\in R^{n\times n}$, where *n* is the number of nodes. $A_{ij}=1$ if $\left\{v_{i},v_{j}\right\}\in E$, $A_{ij}=0$ otherwise.

Among a variety of GNN architectures, we identified four of them to be commonly used in molecular profile analysis: graph convolutional network (GCN),^72^^,^^73^ graph sample and aggregation (GraphSAGE),^74^ graph attention network (GAT),^75^ and graph isomorphism network (GIN).^76^ These architectures iteratively update the representation of a node by aggregating representations of its neighbors, but using different aggregation strategies. The learned node embeddings are used for node label prediction, sample classification (described as graph classification), and edge label prediction.

Graph convolutional approaches can be broadly categorized into two types^77^: spectral based and spatial based. Spectral-based GCNs, which are more widely adopted, use principles from graph signal processing, such as the graph Laplacian and graph Fourier transform, to map the irregular structure of graphs onto a regular Euclidean space for convolution. In contrast, spatial-based GCNs define the convolution operation directly on the graph by utilizing the local neighborhood information.

Defferrard et al.^73^ and Kipf and Welling^72^ proposed spectral-based GCN, which is defined in the spectral domain of the graph Laplacian (Equation 3). Kipf and Welling approximated the spectral convolution using first-order Chebyshev polynomials. This approximation leads to a simplified convolutional operation:

(Equation 7) $$H^{\left[l+1\right]}=\sigma \left({\tilde{D}}^{-\frac{1}{2}}\tilde{A}{\tilde{D}}^{-\frac{1}{2}}H^{\left[l\right]}W^{\left[l\right]}\right)\text{.}$$

where

• ${\tilde{D}}^{-\frac{1}{2}}\tilde{A}{\tilde{D}}^{-\frac{1}{2}}\in R^{n\times n}$ provides a symmetrically normalized adjacency matrix, which makes sure the new features of a node are calculated by its own and the neighbors’ current features. *n* is the number of nodes;
• $\tilde{A}=A+I$ is the adjacency matrix *A* of the graph with added self-connections (i.e., identity matrix *I*);
• $\tilde{D}$ is the degree matrix of $\tilde{\text{A}}$, i.e., a diagonal matrix with ${\tilde{D}}_{ii}=\sum_{j}{\tilde{A}}_{ij}$;
• $H^{\left[l\right]}\in R^{n\times k_{l}}$ is node features at layer *l*, where $k_{l}$ is the feature size at layer *l.* Each row of $H^{\left[l\right]}$ has the feature vector of a node. $H^{\left[0\right]}=X\in R^{n\times k_{0}}$, where $k_{0}$ is the input feature length, i.e., the number of omics modalities analyzed in our case. $i^{\text{th}}$ row of *X* is the node input features $x_{i}$ for the current sample;
• $W^{\left[l\right]}\in R^{k_{l}\times k_{l+1}}$ is the learnable feature transformation weight matrix for layer *l.* Column *j* of $W^{\left[l\right]}$ dictates which linear combination of the current features will contribute to the feature *j* at layer l+1;
• $\sigma \left(\cdot \right)$ is a non-linear activation function, such as ReLU.

This formulation ensures that the new features of each node are calculated by a normalized, weighted sum of its own features and those of its neighbors. $W^{\left[l\right]}$ works like the kernel of the convolution. It is a small matrix shared by all the nodes.

The node embeddings that come out of the last graph convolutional layer can be used to predict a node property, predict a sample property, or a pair of embeddings can be used to predict if there is a certain kind of relation between these nodes.

#### Node label prediction

Node label prediction is a simple neural network task, where node $v_{i}\in V$ has associated label $y_{i}$. The label value can be known or unknown. A neural network trains on the known labels to learn to predict the label of $v_{i}$ from its embedding $h_{i}$ generated by GNN layers. Node labels are typically not omics-sample dependent in the biological domain, such as whether a gene is an oncogene. In this case, node input features are not generated per sample, instead aggregate features are generated per gene using all samples, such as mutation rate, average expression, etc.

Schulte-Sasse et al.^78^^,^^79^ predict cancer-related genes from multi-omics using a GCN on PPI and GGI networks. They generate positive (cancer gene) and negative (not cancer gene) node labels by collecting evidence from multiple cancer gene databases such as Network of Cancer Genes,^80^ COSMIC,^81^ and other resources. They label those genes with high evidence as positive and label the genes with no evidence as negative. Then they predict the labels of the genes that fall in between.

#### Sample label prediction

Sample label prediction task is done using an aggregation (generally concatenation) of node embeddings, generated per sample. A neural network at the end of the architecture learns to map the aggregate embedding to the sample label. For instance, GraphSurv^82^ uses this approach to predict cancer survival from multi-omics. Instead of one large undirected network, they use multiple small networks derived from gene product associations in KEGG pathways. They aggregate node embeddings from the networks to predict survival using a neural network.

**Figure 10:**
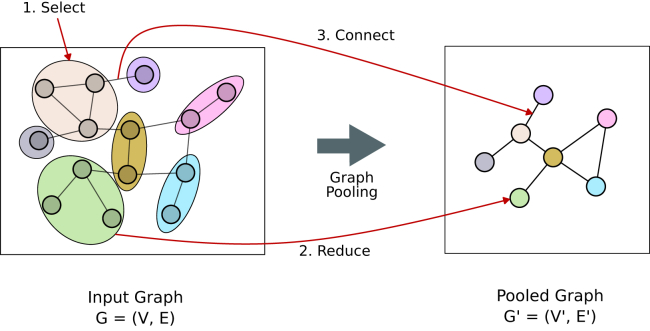
Basic overview of graph pooling

If a very large guide graph is used in sample label prediction, such as the entire known PPI relations, the resulting aggregate node embeddings would be very large due to the high number of nodes on the graph. This makes the algorithm very likely to overfit the data. To address this and reduce the dimensionality, a graph pooling layer can be applied, which aims to downsample the graph by reducing the number of nodes while preserving its essential structural information (Figure 10).

#### Graph pooling

Graph pooling maps a graph *G* to a new graph $G^{\prime }=\left(V^{\prime },E^{\prime }\right)$. There are numerous pooling techniques available, such as: Top-K Pooling,^83^ DiffPool,^84^ SAGPool,^85^ average pooling, MinCut,^86^ etc. The pooling operation can be broadly divided into three operations: selection, reduction, and connection, and they can be written as^87^:

$$\begin{array}{l} S=\text{Select}\left(G\right)={\left\{S_{k}\right\}}_{k=1:K}\\ V^{\prime }={\left\{\text{Reduce}\left(G,S_{k}\right)\right\}}_{k=1:K}\\ E^{\prime }={\left\{\text{Connect}\left(G,S_{k},S_{l}\right)\right\}}_{k,l=1:K} \end{array}$$

Here, *K* is the number of nodes after reducing by the pooling technique. The selection function maps the nodes of the input graph to the nodes of the pooled one. Selection consists of assigning *n* input nodes to *K* sets, $S_{1},S_{2},\ldots S_{K}$, called supernodes. The reduction function computes the node attributes of graph $G^{\prime }$ by aggregating the node attributes of *G* associated with each supernode $S_{k}$. Finally, the connection function determines, for each pair of supernodes $S_{k}$, $S_{l}$, the presence or absence of an edge between the corresponding nodes *k* and *l* in the pooled graph.

GCNs, mostly with pooling, are used for classifying cancers,^88^^,^^89^^,^^90^^,^^91^^,^^92^^,^^93^ predict survival,^94^^,^^95^ classifying the cells in single-cell transcriptomic data,^96^ predicting drug response,^97^ and detecting COPD.^98^

#### GraphSAGE

GraphSAGE^74^ is a spatial-based graph convolution technique. It uses an aggregation function to combine information from the neighboring nodes’ feature vectors, where the aggregation function can be simple such as *mean* or *max*, or it can be complex such as using a neural network. The aggregated feature vector for node *i* at layer *l* is:

(Equation 8) $$h_{N\left(i\right)}^{\left[l\right]}={\text{AGGREGATE}}_{l}\left(h_{j}^{\left[l\right]}\;|\;j\in N\left(i\right)\right)$$

Here, $h_{j}^{\left[l\right]}$ denotes the feature vector of neighbor node *j* at layer *l* and N(i) is the set of the neighbors of *i*. After aggregation, the new feature vector of node *i* is calculated by combining the aggregated neighborhood information with the node’s own feature vector.

(Equation 9) $$h_{i}^{\left[l+1\right]}=\sigma \left(W^{\left[l\right]}\cdot \left(h_{i}^{\left[l\right]}\|h_{N\left(i\right)}^{\left[l\right]}\right)\right)$$

where

• $h_{i}^{\left[l\right]}\in R^{k_{l}}$ is the feature vector of node *i* at layer *l.* $k_{l}$ is the feature size in layer *l*;
• the operation ‖ is called concatenation. It combines two vectors to generate a longer vector;
• $W^{\left[l\right]}\in R^{k_{l+1}\times 2k_{l}}$ is a learnable weight matrix for layer *l*;
• σ(·) is a non-linear activation function.

Instead of applying Equation 8 to all neighbors, GraphSAGE uses random sampling to ensure the number of neighbors for each node remains below a specified threshold, making it scalable for large graphs. Liang et al.^99^ use GraphSAGE to predict cancer survival from transcriptomic data. As for the prior knowledge graph, they use gene-gene relations generated by reducing Reactome pathways with the Graphite^100^ software. Yan et al.^101^ integrate multi-omics using GraphSAGE for survival prediction. They use an interesting guide graph that has multiple nodes per gene, each representing an omics modality. Then they incorporate known gene regulatory relations as edges between mRNA nodes of the transcription factor and its targets, they connect CNV and mRNA nodes of the same genes, and they connect methylation and mRNA nodes of the same genes.

#### Graph attention network

The attention mechanism of transformer architectures has been adapted to GNNs, resulting in GAT proposed by Veličković et al.^75^ GATs introduce the concept of self-attention to graphs, allowing nodes to assign different weights to their neighbors, enabling the model to learn the relative importance of different connections. For a node *i* and one of its neighbors *j*, the normalized attention coefficient $\alpha_{ij}$ is calculated as follows:

(Equation 10) $$\alpha_{ij}^{\left[l\right]}=\frac{\;\exp\;\left(\sigma \left(a^{{\left[l\right]}^{T}}\left[W^{\left[l\right]}h_{i}^{\left[l\right]}\|W^{\left[l\right]}h_{j}^{\left[l\right]}\right]\right)\right)}{\sum_{s\in N^{+}\left(i\right)}\;\exp\;\left(\sigma \left(a^{{\left[l\right]}^{T}}\left[W^{\left[l\right]}h_{i}^{\left[l\right]}\|W^{\left[l\right]}h_{s}^{\left[l\right]}\right]\right)\right)}$$

where

• $h_{i}^{\left[l\right]}\in R^{k_{l}}$ is the feature vector of node *i* at layer *l*, and $k_{l}$ is the size of the node features at layer *l*;
• $W\in R^{k_{l+1}\times k_{l}}$ is a learnable shared linear transformation matrix. $k_{l+1}$ is the feature size at layer l+1;
• $a^{\left[l\right]}\in R^{2k_{l+1}}$ is a learnable weight vector for the attention mechanism at layer *l*;
• exp(x) means x e;
• ‖ is the concatenation operation;
• σ(·) is a non-linear transformation. Authors of GAT used the LeakyReLU function for non-linearity. This is a softer version of ReLU where the negative values are not replaced with a 0 but scaled down by some factor such as 0.2. $\text{LeakyReLU}\left(x\right)=\left\{ \begin{array}{cc} x & \text{if}\;x>0\\ 0.2x & \text{otherwise} \end{array}\right.$.

To increase the model’s expressive power, GATs employ multi-head attention mechanisms, where *D* independent attention heads are used, and their outputs are either averaged (Equation 11) or concatenated.

(Equation 11) $$h_{i}^{\left[l+1\right]}=\sigma \left(\frac{1}{D}\sum_{d=1}^{D}\sum_{j\in N^{+}\left(i\right)}\alpha_{ij}^{{\left[l\right]}_{d}}W^{{\left[l\right]}_{d}}h_{j}^{\left[l\right]}\right)$$

where

• $\alpha_{ij}^{{\left[l\right]}_{d}}$ is the attention coefficient of node *i* for node *j* for the $d^{\text{th}}$ attention head at level *l*;
• $W^{{\left[l\right]}_{d}}$ is the feature transformation matrix for the $d^{\text{th}}$ attention head at level *l*;
• authors of GAT suggest using averaging ($\frac{1}{D}\sum_{d=1}^{D}$) only at the last graph layer. They use concatenation ($\overset{D}{\underset{d=1}{\|}}$) instead in the previous layers.

This multi-head approach allows the model to learn multiple aspects of the influence of neighbors that it needs to attend to. Luo et al.^102^ predict patient responses to neoadjuvant therapy in bladder cancer using transcriptomic data. The model uses two distinct multi-head GAT modules: one for the gene co-expression network and another for the PPI network.

#### GATv2

Brody et al.^103^ propose an improvement to GAT’s attention mechanism’s expressivity and name it GATv2, which uses the following formulation.

(Equation 12) $$\alpha_{ij}^{\left[l\right]}=\frac{\;\exp\;\left(a^{{\left[l\right]}^{T}}\sigma \left(\left[W_{1}^{\left[l\right]}h_{i}^{\left[l\right]}+W_{2}^{\left[l\right]}h_{j}^{\left[l\right]}\right]\right)\right)}{\sum_{s\in N^{+}\left(i\right)}\;\exp\;\left(a^{{\left[l\right]}^{T}}\sigma \left(\left[W_{1}^{\left[l\right]}h_{i}^{\left[l\right]}+W_{2}^{\left[l\right]}h_{s}^{\left[l\right]}\right]\right)\right)}$$

where

• $W_{1},W_{2}\in R^{k_{l+1}\times k_{l}}$ are learnable shared linear transformation matrices. $k_{l+1}$ is the feature size at layer l+1;
• $a^{\left[l\right]}\in R^{k_{l+1}}$ is a learnable weight vector for the attention mechanism at layer *l*;
• σ(·) is LeakyReLU.

Using the attention coefficients $\alpha_{ij}^{\left[l\right]}$ and multi-head attention, the next level features are computed as below.

(Equation 13) $$h_{i}^{\left[l+1\right]}=\sigma \left(\frac{1}{D}\sum_{d=1}^{D}\left(\alpha_{ii}^{{\left[l\right]}_{d}}W_{1}^{{\left[l\right]}_{d}}h_{i}^{\left[l\right]}+\sum_{j\in N\left(i\right)}\alpha_{ij}^{{\left[l\right]}_{d}}W_{2}^{{\left[l\right]}_{d}}h_{j}^{\left[l\right]}\right)\right)$$

where

• $\alpha_{ij}^{{\left[l\right]}_{d}}$ is the attention coefficient of node *i* for node *j* for the $d^{\text{th}}$ attention head at level *l*;
• $W_{1}^{{\left[l\right]}_{d}}$ and $W_{2}^{{\left[l\right]}_{d}}$ are the feature transformation matrix for the $d^{\text{th}}$ attention head at level *l*, for the self and for the neighbors, respectively.

#### Graph link prediction

Inoue et al. propose drGAT,^104^ which uses GATv2 for graph link prediction, where links represent drug sensitivity of cell lines. They construct a graph with cell lines, genes, and drugs as nodes, then connect drugs to their known target genes, connect genes to the cell lines where they are highly expressed, and connect drugs to the cell lines that are sensitive to that drug. They generate drug features by computing drug-drug structural similarity, gene features by their expression similarity in cell lines, and cell features by their drug response similarity. After passing these features through GATv2 layers, they input concatenated pairs of transformed cell features and drug features to an MLP that predicts drug response. drGAT authors use known drug responses both in feature generation (to calculate cell line similarity) and graph generation (to generate drug-cell line edges), which causes concern in performance testing due to data leakage. Data leakage happens when the test data influences the model training, leading to superficially high performance metrics. To address this issue, drGAT masks the test drug-cell line relations (replaces these values with 0) to generate the features and the graph so that the test relations do not influence the training.

GENELink^105^ and GNNLink^106^ use similar approaches to predict links in a GRN using GAT and GCN, respectively. After running GAT layers on node features, GENELink applies a single-layer neural network on the concatenated features of a pair of nodes to infer the link. GNNLink offers a simpler formulation, it takes the inner product of GCN-transformed node features ($h_{i}^{T}h_{j}$) and uses it as a score for link prediction. Dong et al.^107^ provide a review of GRN inference methods that use deep learning models and other statistical methods.

Besides GAT and GATv2, there are other examples of the use of attention mechanism is GNN architectures. For instance, the Graph Transformer^108^ architecture utilizes the complete query, key, and value projection mechanism,^109^ in contrast to the simpler attention mechanism employed by GAT. This is used by Jeong et al.^110^ on a GGI graph to identify molecular biomarkers in eosinophilic asthma subtypes from proteomics, DNA methylation, and transcriptomics.

#### Graph isomorphism network

Xu et al.^76^ address a problem in GCNs: during message passing from a node’s neighbors, the features are transmitted but the topological features of the nodes are not. For instance, if a node’s being a hub node is important for the prediction, then this is lost because it is not encoded in the aggregated features, which are averaged during aggregation. As a solution, they propose the GIN, which uses concepts from a graph isomorphism detection method called the Weisfeiler-Lehman test.^111^ Instead of averaging, GIN takes the sum of its own and neighbors’ features, and transforms it through an MLP for the next layer.

(Equation 14) $$h_{i}^{\left[l+1\right]}={\text{MLP}}^{\left[l\right]}\left(\left(1+\epsilon^{\left[l\right]}\right)\cdot h_{i}^{\left[l\right]}+\sum_{j\in N\left(i\right)}h_{j}^{\left[l\right]}\right)$$

where

• $h_{i}^{\left[l\right]}$ is the feature vector of node *i* at layer *l*;
• ${\text{MLP}}^{\left[l\right]}$ is a multi-layer perceptron applied at layer *l* (a learnable, non-linear transformation);
• $\epsilon^{\left[l\right]}$ is a learnable scalar parameter or a fixed constant, controlling the contribution of the node’s own feature $h_{i}^{\left[l\right]}$.

Pfeifer et al.^112^ use GIN on a PPI graph to predict cancer type from gene expression and DNA methylation. Then they use the method GNN Explainer^113^ to find the influential nodes in predicting each cancer type and name their induced subgraph as disease subnetworks.

The architectures we discussed so far are developed for homogeneous graphs with no node or edge types. Prior knowledge graphs, however, can be heterogeneous such as in signaling networks with different edge types, or detailed process diagrams. Another example is the network that Li and Nabavi^114^ construct for cancer subtype prediction from transcriptomic, CNV, and micro RNA (miRNA) data. They start with a GGI network, add miRNA nodes, and connect miRNAs with their known target genes. Their solution to this heterogeneity is to treat it as homogeneous and apply GCN. But there is this problem: gene nodes have two features on them (mRNA and CNV) while miRNA nodes have only one feature. GCN requires all feature vectors to be the same length in a layer. To fix this, the authors add a dimension increase layer at the beginning of the architecture with two MLPs that take those features and project to a higher-dimensional space to equalize their length. The parameters in these MLPs are learned during the training. The method drGAT (mentioned previously) uses the same strategy to apply GATv2 to a heterogeneous graph where nodes have different length features.

#### Graph heterogeneity

Graph heterogeneity was addressed in some recent architectures such as relational graph convolutional networks (R-GCNs),^115^ heterogeneous graph attention networks,^116^ and heterogeneous graph transformers.^117^ The common idea in these methods is to use type-specific weight matrices in transformations.

For example, R-GCN extends spatial graph convolutions to handle multiple edge types. Such graphs can be defined as G=(V,E,R), where $v_{i}\in V$ is the set of nodes and $\left(v_{i},r,v_{j}\right)\in E$ is the set of edges, where r∈R is an edge type. Each edge type has its own transformation matrix, and messages are passed across nodes taking into account the edge type connecting them.

(Equation 15) $$h_{i}^{\left[l+1\right]}=\sigma \left(\left(\sum_{r\in R}\sum_{j\in N^{r}\left(i\right)}\frac{1}{c_{i,r}}W_{r}^{\left[l\right]}h_{j}^{\left[l\right]}\right)+W_{0}^{\left[l\right]}h_{i}^{\left[l\right]}\right)$$

where

• $h_{i}^{\left[l\right]}$ and $h_{i}^{\left[l+1\right]}$ are features vectors of node *i* at layers *l* and l+1, respectively;
• $N^{r}\left(i\right)$ is the set of neighbors of node *i* connected with edge type *r*;
• $W_{r}^{\left[l\right]}$ is the weight matrix for relation *r* at layer *l*;
• $W_{0}^{\left[l\right]}$ is the weight matrix for self-features;
• $c_{i,r}$ is a problem-specific normalization constant that can be learned or chosen in advance (such as $c_{i,r}=\left|N^{r}\left(i\right)\right|$).

This area, however, remains relatively under-explored compared with the more established research on homogeneous GNNs.

## Discussion

### Model complexity

Directly applying MLP to molecular profiles results in very complex models, meaning the model would contain too many parameters (weights) to work effectively. The strategies we review here reduce this complexity with the help of prior knowledge. For instance, transforming the input into gene set enrichments would shrink the input size when a limited number of gene sets are used. Transforming them into images may not seem like a reduction, but the convolutional layers in a CNN use much fewer parameters (filter weights) than a fully connected layer. By imitating prior knowledge organization in the neural network architecture, we eliminate the vast majority of parameters, resulting in simpler models. The complexity of GNNs depends on the specific architecture. In the case of a GCN, a graph convolution layer uses a small weight matrix for the transformation, which can be used to reduce the size of the feature vector of the nodes.

The success of this reduction in complexity strongly depends on the relevance of the prior knowledge to the prediction task. In all these architectures, the prior knowledge guides the algorithm where to focus. But if the answer lies somewhere else, such as an undiscovered mechanism, then this focus could be detrimental instead of beneficial. This highlights the importance of high-quality and comprehensive models of prior knowledge in molecular biology.

### Interpretability

The “black box” nature of deep neural networks poses challenges for researchers and clinicians seeking to validate and trust model outputs. However, the high-stakes nature of these predictions in clinical settings necessitates a thorough understanding of how these models arrive at their conclusions. In addition to the trust issue, understanding the mechanisms behind a successful prediction provides an opportunity to develop new therapies. For instance, if the short survival of a patient is predicted due to an overactive pathway, targeting that pathway may be considered as part of personalized therapy.

There are already methods developed for neural networks that identify the specific input values with high influence on the results, such as SHAP,^118^ but interpreting them at the input level is generally not easy. Combined with the strategy that uses gene set enrichment scores as input, we can connect biological mechanisms to the predictions. The same thing applies to the image transformation strategy. We have methods developed for CNNs that detect influential regions in an image for the prediction, such as LRP,^119^ SmoothGRAD,^120^ and Grad-CAM.^121^ We can trace back these regions to ontology terms, gene sets, or a set of close genes in an interaction network, each may suggest a biological mechanism. GNNs can be analyzed to identify which connected subgraph influences the results, using methods such as GNN Explainer.^113^ Those protein or gene interactions suggest a mechanism to investigate. The use of prior knowledge in deep learning has great potential to increase its interpretability.

### Limitations

Integration of prior knowledge into deep learning can be useful only if the prior knowledge is relevant to the prediction task. Unfortunately, the knowledge of molecular biology is far from complete, which is the major limitation of using prior knowledge in deep learning. On the other hand, prior knowledge can be too much depending on how it is used. For instance, the number of human gene sets defined in MSigDB is higher than the number of human genes. Using all of it in deep learning may increase the search space instead of decreasing it, hurting the performance. In general, choosing the relevant prior knowledge is an important first step in these strategies, which is also a limitation because it is a manual task.

### Looking ahead

Prior knowledge is modeled in different flavors, focusing on different aspects of known biology and using different levels of complexity (see Box 2). Simpler models typically have a higher coverage of known biology than detailed models. Simpler models are also easier to utilize in machine learning than complex models. Gene sets, hierarchical ontologies, and protein interaction networks are the most frequently used prior knowledge modalities in deep learning due to their simple structure and high coverage. Detailed models, such as directed signaling networks and process models, are harder to build as they typically require manual curation from the literature. But this picture is changing as curators started taking help from large language models.^122^ We anticipate that, in time, the research field will move toward utilizing more detailed models, especially when proteomics becomes more abundant. Proteomics can measure changes in protein modifications, which are only included in detailed models. Utilizing these models, however, requires further architectural innovations in deep learning.

Single-cell omic technologies open a window to cell-to-cell heterogeneity in analyzing tissue samples and cell populations. Compared with bulk measurements, this presents new opportunities for deep learning systems in making predictions as these systems can now see each cell type separately. In addition, we can observe variations of readouts in a specific cell type and identify correlated features. The presence or absence of such correlations may indicate that certain mechanisms are functional or non-functional in the cells, providing new information that can be used in predictions.

## Acknowledgments

This work was supported by the Division of Intramural Research (DIR) of the National Library of Medicine (NLM).

## Declaration of interests

The authors declare no competing interests.

## Supplemental information

Table S1. The list of the reviewed studies
