# Strategies to include prior knowledge in omics analysis with deep neural networks

**DOI:** 10.1016/j.patter.2025.101235

**Published:** 2025-04-04

**Authors:** Kisan Thapa, Meric Kinali, Shichao Pei, Augustin Luna, Özgün Babur

## Main text

(Patterns *6*, 101203; March 14, 2025)

In the originally published version of this article, the authors mentioned “mean-squared error” instead of “squared error” for Equation 6. This has been corrected online. Additionally, several writing and composition errors that do not affect the outcomes of the study have been fixed.

